# LICHEN SCROFULOSORUM: AN IMPORTANT MARKER OF OCCULT TUBERCULOSIS

**DOI:** 10.4103/0019-5154.41656

**Published:** 2008

**Authors:** Naina Dogra, Shazia Shah, Devraj Dogra

**Affiliations:** *From the Department of Dermatology and Venereology, Govt. Medical College, Jammu, India*

**Keywords:** *Lichen scrofulosorum*, *lymphadenitis*, *tuberculid*

## Abstract

Lichen scrofulosorum is a tuberculid that is usually seen in children or young adults. Although a rare occurrence, this tuberculid is an important marker of occult tuberculosis, which may not be detected otherwise. We report here a case of lichen scrofulosorum in a ten year-old boy with typical grouped lichenoid papules on the trunk associated with axillary tuberculous lymphadenitis.

## Introduction

Lichen scrofulosorum is a rare tuberculid that presents as a lichenoid eruption of minute papules in children and adolescents with tuberculosis. The lesions are usually asymptomatic, closely grouped, skin-colored to reddish-brown papules, often perifollicular and are mainly found on the abdomen, chest, back, and proximal parts of the limbs. The eruption is usually associated with a strongly positive tuberculin reaction.^1^

## Case History

A ten year-old boy from a low socio-economic stratum of society presented to us with a generalized papular rash on the trunk and proximal parts of the upper and lower extremities with mild itching since the last two months. There was no history of fever, cough, anorexia, weight loss or any other systemic symptoms. He had received various treatments in the form of potent topical steroids, keratolytics, antibiotics and oral antihistamines in the past but had not shown any improvement. Past and family history was not contributory.

General physical examination revealed a thinly built boy with moderate pallor and matted, nontender, firm, 2 to 1.5 cm-sized lymph nodes in the right axilla. The overlying skin was not involved. No other significant lymphadenopathy or organomegaly was present. Systemic examination of the respiratory, cardiovascular, abdominal and central nervous systems did not reveal any abnormalities.

Cutaneous examination showed multiple, grouped, skin-colored to reddish-brown, follicular and extrafollicular lichenoid papules with mild scaling located on the front and back of the trunk, upper arms, thighs and sides of the face. The lesions were asymptomatic and densely distributed over the upper back. Examination of hair, nails and mucosal surfaces did not reveal any abnormalities.

Laboratory examination indicated anemia with a hemoglobin level of 8 g%, erythrocyte sedimentation rate of 50 mm in the first hour and a reactive Mantoux test with an induration of 18 × 14 mm. Fine needle aspiration cytology from the right axillary lymph node showed a few epitheloid cell clusters. Skin biopsy from a papule on the trunk showed noncaseating, epitheloid cell granulomas in the superficial dermis surrounding a hair follicle. Tubercular bacilli could not be detected on acid-fast staining. Culture for *Mycobacterium tuberculosis* was sterile. Chest radiography and all the other routine investigations were normal.

Antituberculosis therapy was initiated with four drugs - Rifampicin, Isoniazid, Ethambutol and Pyrazinamide for the first two months, followed by Rifampicin and Isoniazid for six months. The patient showed complete clearance of all skin lesions within two weeks of starting the treatment whereas the axillary lymph nodes showed significant reduction in size only after two months of therapy.

## Discussion

Lichen scrofulosorum was first described by Hebra in 1868 as a lichenoid eruption in children and young adults with tuberculosis and strongly positive tuberculin reactions.^1^ The eruption consists of tiny, perifollicular, lichenoid papules arranged in groups. Other dermatoses which have to be differentiated from the eruption include keratosis pilaris, lichen spinulosis, lichen nitidus, pityriasis rubra pilaris and lichenoid sarcoidosis. Follicular papules in keratosis pilaris are usually noninflammatory and present on the upper thighs and arms with little tendency to grouping. Lichen spinulosis usually presents with a spiny process over the lichenoid papule. In lichen nitidus, shiny lichenoid papules are predominantly extrafollicular with characteristic involvement of male genitalia. Pityriasis rubra pilaris and lichenoid sarcoidosis can be differentiated by their characteristic histology.^2^ The pathogenesis of lichen scrofulosorum is considered to be a hematogenous spread of bacilli that are usually not detected in tuberculids as they are present in a fragmented form or have been destroyed by immunological mechanisms.^1^,^2^

The concept of the tuberculid was introduced by Darier in 1896.^1^ A tuberculid is a cutaneous immunological reaction to the presence of an occult tuberculosis in a patient with moderate to high immunity. The main features of tuberculids are a positive tuberculin test, evidence of past or present occult tuberculosis and a good response to antituberculosis therapy.^1^ In the past, many skin disorders were interpreted as tuberculids while presently, only three conditions are considered as true tuberculids: lichen scrofulosorum, papulonecrotic tuberculid and erythema induratum of Bazin. The others such as lupus miliaris disseminata faciei (LMDF) are referred to as pseudotuberculids as they do not respond to antituberculous therapy despite having a tuberculoid histology.^3^

Of the three tuberculids, the incidence of lichen scrofulosorum was found to be the lowest (2%) in a large study conducted in Hong Kong.^4^ This highlights its rarity and significance as an important marker of undetected tuberculosis.^4^ We report here a case of this rare dermatosis for its academic interest.
